# Neutrophils and Neutrophil Extracellular Traps in Hepatic Ischemia–Reperfusion Injury: Molecular Mechanisms and Therapeutic Strategies

**DOI:** 10.3390/ijms27114839

**Published:** 2026-05-27

**Authors:** Furong Xu, Meiyan Wang, Bingxin Wang, Jian Yang, Xiaoqing Qian

**Affiliations:** 1 Department of Emergency, The First Affiliated Hospital of Shihezi University, Shihezi 832000, China; xufurong1@xjshzu.com; 2 School of Medicine, Shihezi University, Shihezi 832000, China; xunyicaoguihua@163.com (M.W.); wangbingxin1@xjshzu.com (B.W.); 3 School of Pharmacy, Shihezi University, Shihezi 832003, China; 4 Key Laboratory of Xinjiang Phytomedicine Resources and Utilization, Ministry of Education, Shihezi 832003, China; 5 Collaborative Innovation Center for Efficient Production and Resource Utilization of Safflower, Institute for Safflower Industry Research of Shihezi University, Shihezi 832003, China

**Keywords:** hepatic ischemia–reperfusion injury (HIRI), neutrophils, neutrophil extracellular traps (NETs), sterile inflammation, targeted therapy

## Abstract

Hepatic ischemia–reperfusion injury (HIRI) is an unavoidable clinical challenge in liver transplantation, major hepatectomy and trauma resuscitation, with no approved specific therapeutics to date. As core innate immune effector cells, neutrophils are the central driver of the sterile inflammatory cascade in HIRI. Centered on the Mac-1-Syk core regulatory axis, this review systematically elaborates the neutrophil recruitment cascade and intracellular signaling network in HIRI, focuses on the sequential formation pathways of neutrophil extracellular traps (NETs) and their multi-dimensional injury mechanisms, and dissects the inflammation–thrombosis amplification crosstalk between neutrophils and multiple hepatic non-parenchymal cells. Furthermore, this review clarifies research controversies of core therapeutic targets, analyzes key translational bottlenecks, and proposes a NETs-centered sequential multi-target combination strategy, providing a solid theoretical basis and clear translational direction for the precise targeted therapy of HIRI.

## 1. Introduction

Hepatic ischemia–reperfusion injury (HIRI) is one of the most common and destructive perioperative complications of complex liver surgeries, including liver transplantation and major hepatectomy, and is also a major cause of morbidity and mortality in patients with severe liver trauma [1]. The hallmark pathological feature of HIRI is the synergy between cellular energy depletion during the ischemic phase and the explosive sterile inflammatory cascade during the reperfusion phase, ultimately leading to extensive hepatocyte necrosis, hepatic microcirculatory disturbance, and irreversible liver function impairment [2]. During this complex pathophysiological process, ischemia-induced hepatocyte death releases various damage-associated molecular patterns (DAMPs), among which high-mobility group box 1 (HMGB1) and mitochondrial DNA (mtDNA) are the most prominent [3,4,5,6]. These DAMPs rapidly activate resident Kupffer cells (KCs) and liver sinusoidal endothelial cells (LSECs), thereby triggering an uncontrolled inflammatory cascade characterized by massive neutrophil infiltration.

**Figure 1:**
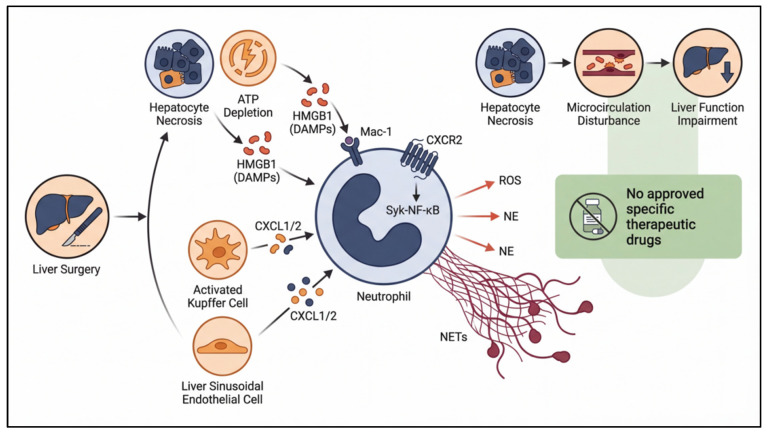
Hepatic ischemia–reperfusion injury (HIRI), a major perioperative complication of liver surgery with no approved specific therapies, is initiated by ischemia–reperfusion-induced sterile inflammation: DAMPs activate resident liver cells to recruit neutrophils, which induce tissue damage via oxidative stress, proteases, and NETs, causing liver dysfunction. This figure depicts the core cascade and translational challenges of targeted therapies.

Neutrophils are the most abundant leukocyte subset in the peripheral circulation. Extensive evidence from preclinical animal models has unequivocally demonstrated that excessive neutrophil activation and aberrant hepatic infiltration are core pathogenic drivers of HIRI. Indeed, neutrophil depletion or selective functional inhibition significantly reduces hepatic necrosis and improves liver function [7,8,9]. However, to date, all neutrophil-targeted therapeutic strategies have failed to translate into clinical practice. The fundamental reasons lie in three key unresolved scientific controversies and knowledge gaps: first, the significant discrepancy between the classical leukocyte recruitment paradigm and the organ-specific regulatory mechanisms dictated by the unique hepatic sinusoidal microenvironment, particularly the non-redundant role of integrin Mac-1 in the neutrophil adhesion cascade and its molecular mechanisms, which remain incompletely elucidated [10,11]; second, the delicate balance between the physiological antimicrobial defense function of neutrophils and their pathological tissue-damaging effects remains poorly characterized, and the high risk of severe infections associated with systemic neutrophil inhibition severely limits clinical application; third, the time-dependent pathogenic role of neutrophil extracellular traps (NETs)—a major research focus in the field of sterile inflammation over the past decade—as well as the optimal therapeutic window for targeted intervention and long-term safety, remain highly controversial [12,13,14] (Figure 1).

Against this background, this review provides a comprehensive and critical analysis of the complete pathological cascade of neutrophils in HIRI, from recruitment and activation to the execution of damaging effects. We focus on the core roles of key molecules and signaling pathways, critically analyze the roots of conflicting results among different studies, comprehensively dissect the translational bottlenecks that have hindered the development of neutrophil-targeted therapies, and finally propose promising future research directions and actionable clinical translation strategies.

## 2. The Neutrophil Recruitment Cascade in Hepatic Ischemia–Reperfusion Injury

**Figure 2:**
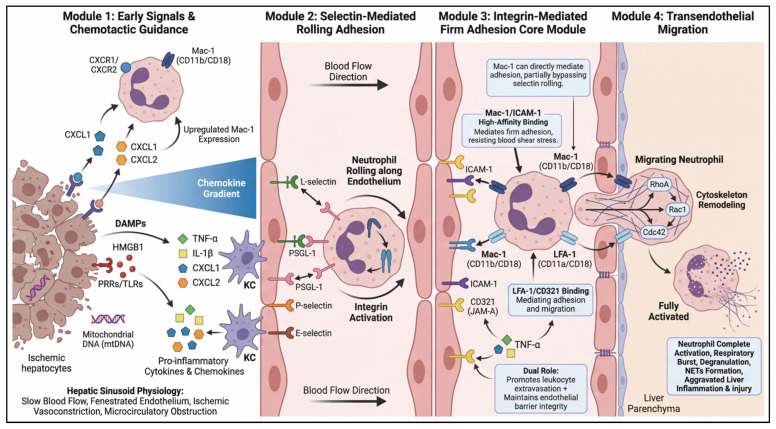
Neutrophil recruitment cascade in hepatic ischemia–reperfusion injury (HIRI). This schematic illustrates the stepwise molecular mechanism of neutrophil recruitment in HIRI, presented in strict temporal and spatial order based on the hepatic sinusoid microenvironment. It includes four core modules: (1) Early signal initiation: DAMPs from necrotic hepatocytes activate liver sinusoidal endothelial cells (LSECs) and Kupffer cells (KCs), which secrete chemokines and pro-inflammatory cytokines to prime circulating neutrophils; (2) Selectin-mediated rolling adhesion: P/E-selectin on activated LSECs mediates neutrophil rolling along the endothelium, triggering integrin affinity switch; (3) Integrin-mediated firm adhesion: High-affinity Mac-1/ICAM-1 and LFA-1/CD321 binding mediates stable neutrophil adhesion; (4) Transendothelial migration: Neutrophils remodel cytoskeleton via Rho GTPases, cross the endothelial barrier into liver parenchyma, and trigger subsequent inflammatory tissue injury.

Neutrophil recruitment to ischemic liver tissue is a prerequisite for their subsequent pathogenic effects. This process is not a simple linear sequence of chemotaxis, adhesion, and migration, but rather a highly ordered, multi-step cascade tightly regulated by various molecular mediators within the unique hepatic sinusoidal microenvironment (Figure 2).

### 2.1. Initiation of Chemotactic Signals: The Dominant Role of the CXCL1/2-CXCR2 Axis

Liver sinusoidal endothelial cells (LSECs) and Kupffer cells (KCs) are the primary sentinel cells that detect DAMPs in the liver, jointly forming the first line of defense for inflammatory signal recognition and amplification. In the early phase of reperfusion, LSECs directly sense circulating DAMPs via surface pattern recognition receptors (PRRs) and become rapidly activated. LSEC injury itself can trigger KC and neutrophil activation, hepatic sinusoid constriction, and endothelial dysfunction, thereby establishing the structural and signaling foundation for subsequent neutrophil recruitment [15].

Upon DAMP recognition, activated KCs secrete large amounts of CXCL1 and CXCL2, the core chemokines driving neutrophil chemotaxis in HIRI. These chemokines bind to CXCR2, which is constitutively highly expressed on the neutrophil surface, forming a steep chemotactic concentration gradient across the liver sinusoidal endothelium. Resting neutrophils in the circulation detect this CXCL1/2 gradient via their surface CXC chemokine receptors 1 and 2 (CXCR1/CXCR2), initiating an intracellular G protein-coupled receptor (GPCR)-mediated signaling cascade. This signal rapidly converts neutrophils from a resting state to an activated phenotype, preparing them for subsequent adhesion and transendothelial migration [16].

In addition to producing chemokines, KC-derived TNF-α and IL-1β exert dual pro-inflammatory effects: they not only further enhance chemokine synthesis by LSECs and KCs themselves, but also upregulate Mac-1 expression on the neutrophil surface [17]. This synergistic action both increases the adhesive capacity of neutrophils to endothelial cells and enhances their sensitivity to chemotactic signals. Both gene knockout and pharmacological inhibition studies consistently show that CXCR2 deficiency significantly reduces neutrophil infiltration, alleviates hepatic necrosis, and improves liver function after HIRI in mice [18,19,20,21].

However, it must be noted that the chemokine system exhibits extensive functional redundancy. Blockade of the CXCL1/2-CXCR2 axis alone cannot completely eliminate neutrophil recruitment, which is the main reason why, despite remarkable efficacy in preclinical animal models, CXCR2 inhibitors (such as reparixin) have progressed slowly toward clinical translation [22]. Although the CXCL1/2-CXCR2 axis is the primary pathway mediating neutrophil chemotaxis in HIRI, the CXCL12/CXCR4 axis plays a critical role in other biological processes, particularly hematopoietic stem cell homing. Although studies on the role of CXCL12/CXCR4 in HIRI remain limited compared with CXCR2, given that CXCL12 is expressed by multiple liver stromal cell populations [23], this pathway may serve as an alternative or auxiliary chemotactic axis regulating the localization or retention of neutrophils in specific hepatic microdomains, representing an important direction for future research. Moreover, common clinical pathological conditions such as hyperglycemia and hyperosmolarity can enhance neutrophil chemotactic sensitivity by upregulating chemokine receptor expression—a phenomenon largely overlooked in existing studies but potentially a major source of significant heterogeneity in clinical patient outcomes [24,25].

### 2.2. The Core of the Adhesion Cascade: Non-Redundant Regulatory Functions of Mac-1 Integrin

The distinctive features of the hepatic sinusoid—low blood flow velocity and fenestrated endothelium—make it highly susceptible to ischemic injury during HIRI. LSEC injury induces sinusoidal constriction and microcirculatory disturbance, further reducing blood flow velocity. Simultaneously, upregulation of adhesion molecules and disruption of endothelial barrier integrity enhance the ability of hepatic vessels to capture circulating neutrophils, creating a favorable environment for the adhesion cascade [26].

In the very early phase of reperfusion, the initial steps of neutrophil recruitment are mediated by selectin family adhesion molecules. KC-derived TNF-α induces LSECs to express intercellular adhesion molecule-1 (ICAM-1) and P-selectin, promoting neutrophil rolling and tethering [27]. Reperfusion rapidly upregulates P-selectin and E-selectin on the LSEC surface, while neutrophils constitutively express L-selectin [28]. Low-affinity interactions between selectins and their neutrophil ligands (most importantly P-selectin glycoprotein ligand-1, PSGL-1) mediate neutrophil rolling along the endothelial surface. This rolling process reduces neutrophil velocity, providing sufficient time for subsequent integrin activation and firm adhesion. During rolling, the continuously strengthening chemotactic signal induces a conformational change in integrins from a low-affinity to a high-affinity state, enabling stable adhesion [29,30].

Importantly, the hepatic sinusoidal microenvironment possesses unique features significantly different from the classical leukocyte adhesion cascade. LSECs constitutively express ICAM-1 under physiological conditions, and its expression is further markedly upregulated under pathological conditions. Consequently, neutrophil capture in the liver may partially bypass the classical selectin-mediated rolling step and rely mainly on Mac-1-mediated firm adhesion [31]. Mac-1 (CD11b/CD18) is the prototypic member of the β2 integrin family, a heterodimeric glycoprotein formed by non-covalent association of α (CD11b) and β (CD18) subunits. Mac-1 not only functions as an adhesion molecule but also serves as a multi-functional signal transduction platform. On resting neutrophils, Mac-1 adopts a bent, low-affinity conformation. Chemotactic signals trigger “inside-out” signaling, inducing extension of the Mac-1 ectodomain and exposure of ligand-binding sites, leading to a sharp increase in binding affinity [17,27]. Activated Mac-1 then engages in high-affinity interactions with ICAM-1, which is significantly upregulated on liver sinusoidal endothelial cells after reperfusion. This firm interaction enables neutrophils to resist blood flow shear stress and stably arrest on the vessel wall, representing a critical transition from dynamic rolling to static adhesion [32].

Numerous studies have confirmed the central role of Mac-1 in HIRI: the expression of Mac-1 and ICAM-1 in the liver is significantly upregulated after HIRI [33]; administration of anti-Mac-1 or anti-CD18 monoclonal antibodies, as well as genetic knockout of Mac-1/CD18 in mice, effectively reduces hepatic neutrophil infiltration and alleviates liver injury [7,34]. Moreover, simvastatin has been shown to ameliorate hyperlipidemia-exacerbated HIRI by reducing Mac-1 expression and inhibiting NET formation [35].

### 2.3. Dual Regulation of Transendothelial Migration: The Functional Paradox of CD321

Following firm adhesion, neutrophils must traverse the endothelial barrier either through intercellular junctions (paracellular migration) or directly through the endothelial cell body (transcellular migration) and penetrate the basement membrane to reach the hepatic parenchyma. This is an active process requiring extensive cytoskeletal reorganization and dramatic morphological changes in neutrophils. Neutrophils extend pseudopodia to probe for weak points at endothelial cell junctions. Mac-1 continues to play a critical role during this stage, not only maintaining the physical connection between neutrophils and endothelial cells but also guiding migration direction through interactions with additional endothelial ligands such as junctional adhesion molecules (JAMs) and ICAM-2. Studies have demonstrated that Mac-1-deficient neutrophils exhibit significantly impaired transendothelial migration capacity, confirming its essential role in guiding neutrophils across the endothelial barrier [36,37].

CD321, also known as junctional adhesion molecule-A (JAM-A), is a member of the immunoglobulin superfamily. Under physiological conditions, CD321 is localized to endothelial tight junctions, where its main function is to maintain endothelial barrier integrity and cell polarity. Within 1 h after hepatic IRI, CD321 expression on LSECs is rapidly upregulated and remains elevated for 45 min after reperfusion, suggesting its active involvement in the early inflammatory response [10,38].The pro-inflammatory function of CD321 is primarily mediated through its interaction with lymphocyte function-associated antigen-1 (LFA-1, CD11a/CD18) on the neutrophil surface. This CD321-LFA-1 interaction mediates both firm adhesion and transendothelial migration of neutrophils, and is particularly critical during the early reperfusion phase (within the first 2 h) [39]. Experimental evidence shows that intraportal injection of the anti-CD321 monoclonal antibody 90G4 significantly inhibits early neutrophil infiltration, reduces serum transaminase levels and hepatocyte apoptosis, and decreases hepatic production of IL-6 and TNF-α [10].

Transendothelial migration also involves extensive cytoskeletal remodeling within neutrophils, which is tightly regulated by the activity of Rho family GTPases, including RhoA, Rac1, and Cdc42 [40,41]. Once neutrophils successfully transmigrate into the hepatic parenchyma, they execute their effector functions, leading to progressive tissue damage.

## 3. Key Intracellular Signaling Pathways Regulating Neutrophil Activation and Migration

**Figure 3:**
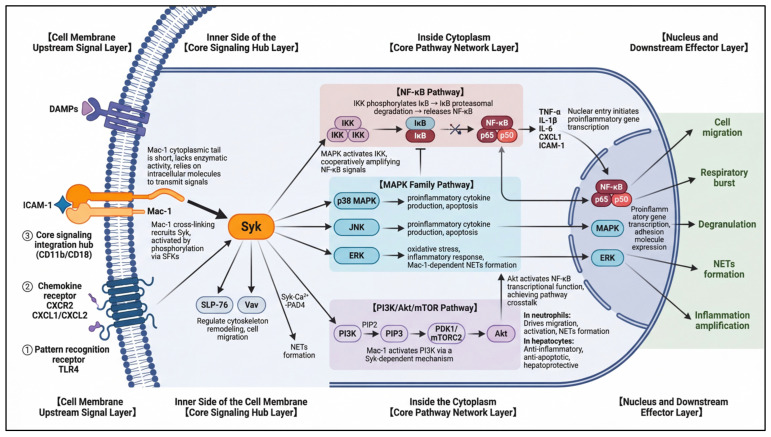
Key intracellular signaling pathways of neutrophil activation and migration in HIRIThis schematic illustrates the full signaling cascade driving neutrophil activation and migration in hepatic ischemia–reperfusion injury (HIRI). Centered on the Mac-1-Syk core axis, it presents upstream membrane receptors, three core downstream pathways (NF-κB, MAPK, PI3K/Akt), their crosstalk, and final pro-inflammatory biological effects including NETs formation, which are core drivers of HIRI progression.

Upon receiving upstream signals from DAMPs and PRRs, neutrophils activate a complex intracellular signaling network that coordinates all of their cellular effector responses, including gene expression, chemotaxis, respiratory burst, and degranulation. The chemotactic, adhesive, and effector functions of neutrophils are precisely regulated by this interconnected signaling network, in which the Mac-1-Syk axis has emerged as a central integration hub. Mac-1 itself has a short cytoplasmic tail and lacks intrinsic enzymatic activity; thus, all extracellular adhesion signals transmitted through Mac-1 rely entirely on spleen tyrosine kinase (Syk)-mediated “outside-in” signaling [42,43]. Activated Syk subsequently activates three major downstream signaling cascades: the mitogen-activated protein kinase (MAPK), phosphatidylinositol 3-kinase (PI3K)/Akt, and nuclear factor-κB (NF-κB) pathways, which coordinately regulate neutrophil activation and pathogenic effector functions. A fundamental limitation of existing studies is that most have relied on systemic inhibitors or global knockout models, which cannot distinguish the cell-specific effects of these pathways. This knowledge gap is a major reason for the frequent occurrence of off-target toxicity issues during clinical translation (Figure 3).

### 3.1. The Central Signaling Hub: Non-Redundant Regulatory Functions of Syk Kinase

Syk is a non-receptor tyrosine kinase that plays a central role in immune cell signaling, most notably in B-cell receptor and Fcγ receptor signaling. Accumulating evidence has established Syk as a major and indispensable downstream effector of integrin (particularly Mac-1) signaling. Mac-1 crosslinking recruits Syk to the plasma membrane, where it is phosphorylated and activated by Src family kinases (SFKs). Activated Syk then phosphorylates a range of downstream substrates, including adaptor proteins (SLP-76, Vav) and effector enzymes, to regulate cytoskeletal remodeling. This positions Syk as a key molecular bridge linking Mac-1 to multiple critical downstream pathways, including PI3K/Akt and MAPK [44,45].

In the context of HIRI, Syk provides the necessary signaling environment for NET formation by regulating cytoskeletal rearrangement, reactive oxygen species (ROS) generation, and inflammatory cytokine release [46,47]. Mechanistically, rapid activation of Syk in neutrophils after HIRI promotes NET formation through two distinct pathways: first, by promoting nuclear translocation of pyruvate kinase M2 (PKM2) and subsequently upregulating STAT3 phosphorylation; second, by activating the NLRP3 inflammasome in macrophages, enhancing IL-1β secretion, which further amplifies neutrophil activation and NET release [48]. Moreover, binding of P-selectin to PSGL-1 promotes NET formation via the Syk-Ca^2+^-PAD4 axis [45], confirming Syk as a convergence node for multiple signaling pathways that regulate NETosis.

### 3.2. Effects of the Three Major Syk Downstream Pathways

#### 3.2.1. The Mitogen-Activated Protein Kinase (MAPK) Family Pathways

The MAPK family is another set of key signaling cascades that are strongly activated during HIRI. These evolutionarily conserved kinases convert extracellular stimuli into intracellular responses and are critical mediators of inflammation, stress responses, and cell migration. After reperfusion, MAPK pathways undergo rapid and sustained phosphorylation, indicating their central role in HIRI pathogenesis [49,50]. As direct downstream targets of Syk, the three major MAPK subfamilies—p38 MAPK, c-Jun N-terminal kinase (JNK), and extracellular signal-regulated kinase (ERK)—coordinately regulate neutrophil migration, cytokine production, and NET formation.

Activation of p38 and JNK primarily mediates pro-inflammatory effects and apoptosis. Pharmacological inhibition of p38 MAPK reduces neutrophil responsiveness to chemokines and impairs their migratory ability [51]. Mechanistic studies have identified several upstream regulators of this axis: rhomboid domain-containing protein 5 homolog 2 (Rhbdf2) exacerbates HIRI by activating the TAK1-JNK/p38 pathway [52]; conversely, 6-gingerol exerts anti-inflammatory and anti-apoptotic effects by modulating MKP5-mediated p38/JNK signaling [53]. Similarly, human liver transplantation peptide 1 (HLTP1) protects against HIRI by inhibiting JNK-mediated hepatocyte apoptosis [54], while nepetalactone B (NB) alleviates liver injury by suppressing the p38/JNK cascade [55]. Notably, hepatic IRI and activation of the ASK1/JNK/p38 MAPK axis are associated with reduced expression of dual-specificity phosphatase 12 (DUSP12) [56]. Therefore, preventing DUSP12 downregulation or enhancing its activity may represent a novel therapeutic strategy to inhibit the ASK1/JNK/p38 pathway and alleviate liver injury.

The ERK signaling pathway regulates neutrophil oxidative stress and inflammatory responses. Receptor activity-modifying protein 1 (RAMP1), a member of the GPCR adaptor protein family, protects against HIRI by inhibiting ERK and YAP phosphorylation [57]. In contrast, METTL3 deficiency exacerbates HIRI in mice by activating the MAPK signaling pathway [58], identifying METTL3 as a potential therapeutic target. Moreover, in ischemic organ injury, Mac-1 has been shown to mediate NET formation through an ERK phosphorylation-dependent mechanism [59].

#### 3.2.2. The Phosphoinositide 3-Kinase (PI3K)/Akt Pathway

The PI3K/Akt pathway is the key downstream signaling cascade of the Mac-1-Syk axis [60], and serves as a core regulator of neutrophil activation, migration and effector functions in the pathological process of HIRI [61,62]. Class I PI3Ks are the most extensively studied subtype in the field of inflammatory diseases, and are further divided into class IA (PI3Kα, PI3Kβ, PI3Kδ) and class IB (PI3Kγ) isoforms. Among them, PI3Kγ and PI3Kδ are predominantly expressed in innate immune cells including neutrophils, while PI3Kα and PI3Kβ are widely distributed in parenchymal cells such as hepatocytes, which lays a molecular basis for the cell-specific functions of this pathway [63].

Mac-1 activation triggers the rapid activation of class I PI3Ks through two Syk-dependent pathways: first, activated Syk directly phosphorylates the regulatory subunit of PI3K to induce its activation; second, Syk-mediated phosphorylation of adaptor proteins (such as SLP-76 and Vav) recruits PI3K to the plasma membrane, further amplifying its activation [64]. Once activated, PI3K catalyzes the conversion of phosphatidylinositol-4,5-bisphosphate (PIP2) to phosphatidylinositol-3,4,5-trisphosphate (PIP3) on the inner leaflet of the plasma membrane. PIP3 acts as a lipid second messenger, recruiting proteins containing pleckstrin homology (PH) domains—most notably the serine/threonine kinase Akt and its upstream activating kinase 3-phosphoinositide-dependent kinase 1 (PDK1)—to the cell membrane. Akt is then fully activated through sequential phosphorylation at Thr308 by PDK1 and Ser473 by mammalian target of rapamycin complex 2 (mTORC2), thereby completing the transduction of integrin-derived signals to downstream effector molecules.

In neutrophils, the activated PI3K/Akt pathway exerts non-redundant regulatory effects on multiple key pathological processes in HIRI: it drives cytoskeletal rearrangement and directed chemotaxis of neutrophils via regulation of small GTPases, promotes the high-affinity conformational switch of Mac-1 to enhance firm adhesion, mediates NADPH oxidase-dependent respiratory burst and ROS production, and acts as a key upstream signaling to promote the formation of NETs [63]. Consistent with these regulatory functions, preclinical studies have confirmed that the PI3K/Akt pathway in circulating neutrophils is significantly and continuously activated during the early reperfusion phase of HIRI, which is positively correlated with the degree of neutrophil infiltration in the liver and the severity of hepatic injury [65].

#### 3.2.3. The Nuclear Factor-κB (NF-κB) Pathway

Nuclear factor-κB (NF-κB) is a family of transcription factors that function as the “master switch” for the initiation and maintenance of inflammatory responses. In resting cells, NF-κB is sequestered in the cytoplasm bound to inhibitory κB (IκB) proteins. Upon stimulation by pro-inflammatory mediators such as TNF-α or DAMPs, the upstream IκB kinase (IKK) complex is activated, phosphorylating IκB and targeting it for proteasomal degradation [66]. The released NF-κB then translocates to the nucleus, where it initiates transcription of a vast array of pro-inflammatory genes, including cytokines (TNF-α, IL-1β, IL-6), chemokines, and adhesion molecules. In resting cells, NF-κB is bound to inhibitory κB (IκB) proteins and sequestered in the cytoplasm. Upon stimulation by pro-inflammatory mediators (such as TNF-α or DAMPs), the upstream IκB kinase (IKK) complex is activated, phosphorylating IκB and targeting it for proteasomal degradation. The released NF-κB then translocates to the nucleus and initiates the transcription of numerous pro-inflammatory genes, including cytokines (TNF-α, IL-1β, IL-6), chemokines, and adhesion molecules [67].

Although often considered an independent inflammatory amplification loop, NF-κB is indirectly activated by Syk through the PI3K/Akt and MAPK pathways. This creates a positive feedback loop coupling NF-κB-mediated gene transcription to the Mac-1 signaling network, sustaining and amplifying the inflammatory response [68]. During HIRI, DAMPs activate the NF-κB pathway through TLR4 receptors, which has two major consequences: first, it promotes the release of chemokines and cytokines from Kupffer cells, enhancing neutrophil recruitment and activation; second, it directly upregulates pro-inflammatory gene expression in neutrophils themselves, enhancing their pathogenic effector functions [69]. Moreover, NF-κB activation is closely associated with apoptosis, further promoting hepatocyte death. Pharmacological inhibition of NF-κB using specific inhibitors (such as Ac2-26) or agents that enhance liver regeneration has been shown to significantly reduce inflammation and tissue injury in HIRI models [70,71].

However, clinical translation of NF-κB inhibitors faces the same fundamental challenge as the other pathways described above: NF-κB is a core transcription factor for host immune defense. Systemic NF-κB inhibition carries a substantial risk of severe infections. Furthermore, NF-κB also regulates hepatocyte survival [72], and non-specific inhibition may paradoxically exacerbate hepatocyte injury.

### 3.3. Crosstalk and Integration Between Signaling Pathways

The downstream signaling pathways described above do not function independently; instead, they engage in extensive bidirectional crosstalk, forming a complex and highly integrated regulatory network. This network architecture explains why single-pathway inhibitors often fail to completely eliminate neutrophil activation and why therapies that show remarkable efficacy in preclinical animal models frequently disappoint in clinical trials.

Akt, the key effector molecule of the PI3K/Akt pathway, activates the transcriptional function of NF-κB through both IKK-dependent and p38 MAPK-dependent mechanisms [73]. The PI3K/Akt/mTOR pathway further amplifies inflammation by activating NF-κB signaling and promoting the production of pro-inflammatory mediators [74]. This positive regulation ensures that once the Mac-1-Syk axis is activated, it synergistically enhances NF-κB-mediated gene transcription to sustain the inflammatory response. Importantly, this crosstalk is also cell-specific: while PI3K/Akt signaling promotes inflammation in neutrophils, it exerts anti-inflammatory, antioxidant, anti-apoptotic, and autophagy-regulatory effects in hepatocytes [75], further highlighting the complexity of pathway regulation in HIRI.

MAPK and NF-κB pathways are also intimately interconnected in HIRI, and can be simultaneously activated by lipid metabolites [76]. Arachidonate 12-lipoxygenase (ALOX12), which is upregulated in the liver following IRI, induces inflammation through activation of both MAPK and NF-κB pathways [77]. Cordycepin pretreatment attenuates HIRI by modulating the MAPK/NF-κB axis, inhibiting inflammation, apoptosis, and autophagy [78]. Mechanistically, MAPK pathways activate NF-κB through phosphorylation of IKK. Rap1 has been identified as a novel regulator of NF-κB signaling that promotes IKK-mediated p65 phosphorylation and subsequent NF-κB target gene expression. Rap1 knockout inhibits activation of both NF-κB and MAPK pathways in a mouse HIRI model and in primary neutrophils [79]. Furthermore, activation of farnesoid X receptor (FXR) inhibits TLR4 and its downstream MAPK and NF-κB pathways, reducing apoptosis and inflammation in HIRI [80]. Consistent with these findings, targeting the MAPK/NF-κB axis has been shown to attenuate inflammation and oxidative stress in multiple other models of ischemia–reperfusion injury [81,82].

## 4. Neutrophil Extracellular Traps (NETs) in Hepatic Ischemia–Reperfusion Injury

### 4.1. Temporal Formation Pathways of NETs

In 2004, Brinkmann et al. first described a novel form of neutrophil death in response to pathogens, termed NETosis. During NETosis, neutrophils actively release their chromatin DNA, which is decorated with histones, neutrophil elastase (NE), myeloperoxidase (MPO), and other granular proteins, forming web-like structures known as neutrophil extracellular traps (NETs) [83]. Physiologically, NETs efficiently trap and eliminate bacteria, fungi, and other pathogens. The major components of NETs include: (1) a DNA backbone that provides a physical barrier and adhesion platform; (2) histones, particularly H3 and H4, which not only serve as structural proteins but also exert direct cytotoxic, pro-inflammatory, and pro-coagulant activities; and (3) granular proteins such as NE and MPO, which participate in NET formation and function as effector molecules mediating antimicrobial and tissue-damaging effects [84].

**Figure 4:**
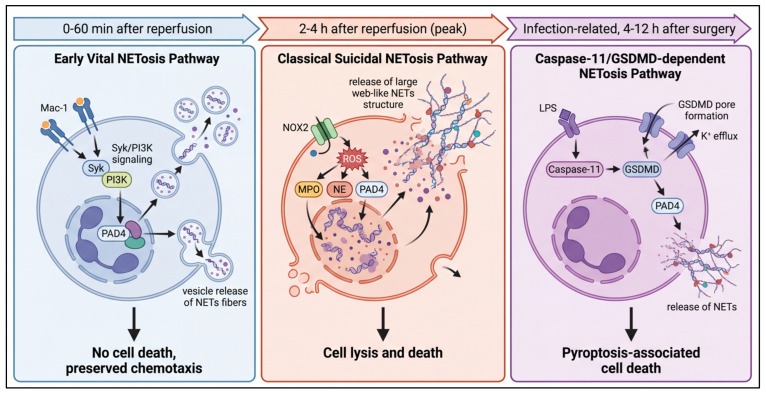
Timeline of three distinct NETosis pathways in hepatic ischemia–reperfusion injury (HIRI). This Biorender-style schematic illustrates three neutrophil extracellular traps (NETs) formation (NETosis) pathways in HIRI via a left-to-right chronological timeline: (1) early vital NETosis (0–60 min post-reperfusion, triggered by Mac-1 integrin activation, no neutrophil death, drives hepatic sinusoidal microcirculation disturbance); (2) classical suicidal NETosis (peaks at 2–4 h post-reperfusion, triggered by NOX-dependent ROS burst and DAMPs, causes neutrophil lysis, core driver of hepatocyte necrosis); (3) infection-related caspase-11/GSDMD-dependent NETosis (4–12 h post-surgery, triggered by bacterial LPS, exacerbates HIRI via pyroptosis-NETosis crosstalk).

However, under sterile inflammatory conditions such as HIRI, NET formation loses its physiological defensive significance and instead becomes a potent endogenous damage signal that drives and amplifies pathological processes [12]. Extensive evidence demonstrates abundant NET formation in both HIRI mouse models and liver tissues from clinical patients, with NET levels closely correlating with the severity of liver injury [85,86]. Importantly, NET formation in HIRI is not mediated by a single pathway but rather by the synergistic action of three temporally and contextually distinct pathways that sustain pathological NET release (Figure 4):

1. Early rapid vital NETosis pathway: Core time window 0–60 min after reperfusion. Activated platelets release P-selectin, which binds to PSGL-1 on the neutrophil surface. This interaction triggers intracellular calcium release and PAD4 activation. This process is completed within minutes, is independent of NADPH oxidase, and leads to rapid NET release via a NOX/ROS-independent vesicular transport pathway. Vital NETosis is the primary cause of microcirculatory dysfunction in early reperfusion [87];
2. Mid-to-late classical suicidal NETosis pathway: Peaks at 2–4 h after reperfusion. Triggered by oxidative stress, this pathway proceeds via a NOX-ROS-dependent PKC-Raf-MEK-ERK cascade, promoting NE translocation to the nucleus, inducing chromatin decondensation, and ultimately leading to plasma membrane rupture and NET release, causing large-scale liver tissue injury [88];
3. Infection-associated Caspase-11/GSDMD-dependent NETosis pathway: Significantly activated when complicated by endotoxemia. It mediates NET release through NLRP3 inflammasome activation and gasdermin D (GSDMD) pore formation, promoting and amplifying inflammatory injury throughout the disease course [89,90].

The sequential activation of these three pathways explains why single-pathway inhibitors (e.g., antioxidants alone) often fail to completely abrogate NET formation, representing a core reason for the limited efficacy of single-target therapies in preclinical studies. Notably, PAD4, a calcium-dependent enzyme that catalyzes the conversion of arginine residues to citrulline residues (citrullination), emerges as a common key node across all three pathways. During NETosis, activated PAD4 translocates to the nucleus and specifically citrullinates histone H3 and H4 residues, mediating chromatin decondensation [91]. As the critical executioner enzyme for NET formation, PAD4 has been extensively validated in preclinical models: both PAD4 genetic ablation and pharmacological inhibition (using Cl-amidine or GSK484) significantly reduce levels of citrullinated histone H3 (CitH3, a specific NET marker) in the liver and attenuate hepatic tissue damage and inflammation [92].

### 4.2. Multidimensional Tissue Injury Mechanisms Mediated by NETs

Once formed, NETs become a central node of the inflammation–thrombosis amplification loop, mediating liver tissue injury through three synergistic mechanisms and establishing a self-sustaining vicious cycle:

1. Direct disruption of the liver sinusoidal endothelial barrier: Histones H3/H4 on NETs directly induce endothelial cell membrane perforation, leading to calcium overload and necrosis. Additionally, histones act as DAMPs to activate TLR4/TLR9 receptors, triggering the NF-κB and MAPK inflammatory pathways, upregulating the expression of adhesion molecules and pro-inflammatory cytokines, and recruiting more neutrophils. This further disrupts endothelial barrier integrity, forming a positive feedback loop of endothelial injury and NET release [93].
2. Induction of microcirculatory dysfunction and no-reflow phenomenon: The negatively charged DNA backbone of NETs activates coagulation factor XII, while histones induce tissue factor expression and platelet activation. Moreover, NETs themselves provide a physical scaffold for platelet and fibrin deposition, jointly promoting microthrombus formation within the hepatic sinusoids. Widespread microvascular obstruction leads to persistent tissue ischemia despite restored blood flow, the so-called “no-reflow phenomenon”, which is a major cause of irreversible progression of HIRI [7,94].
3. Indirect mediation of extensive hepatocellular necrosis: The combined effects of NET-mediated endothelial barrier disruption, cytokine storm, and oxidative stress burst, superimposed on persistent perfusion impairment caused by microthrombosis, ultimately lead to widespread hepatocyte apoptosis and necrosis. Additionally, NET-bound NE and MPO retain sustained enzymatic activity, degrading extracellular matrix and cell membrane proteins to further amplify hepatocellular injury [95].

### 4.3. Endogenous Clearance Mechanisms of NETs

Under physiological conditions, NETs can be cleared by two pathways: ① endogenous DNase I (mainly synthesized by the liver and kidneys) degrades the DNA backbone; ② macrophages engulf intact NETs and their degradation products through efferocytosis. In HIRI, endogenous DNase I activity is significantly inhibited by oxidative stress, and the phagocytic function of Kupffer cells is impaired, leading to sustained NET accumulation and amplified injury [96]. This mechanistic defect explains why exogenous DNase I supplementation effectively alleviates HIRI.

### 4.4. Synergistic Amplification Effects of Proteases and Oxidative Stress

MPO-mediated oxidative stress and NE-mediated proteolysis are not only important components of NETs but also upstream drivers of NET formation, forming a synergistically amplified damage loop.

Neutrophil granules store a variety of potent proteolytic enzymes, including NE and cathepsin G. Upon activation, neutrophils release these proteases through degranulation. These enzymes exhibit broad substrate specificity, degrading extracellular matrix components such as elastin, collagen, and proteoglycans, disrupting liver tissue architecture, and attacking membrane proteins of hepatocytes and endothelial cells to induce cell lysis. NE release is a hallmark of HIRI, and NE inhibition has been consistently shown to attenuate liver injury. Recent studies have further revealed that NE also indirectly amplifies inflammation and thrombosis by degrading anti-inflammatory factors and activating coagulation pathways. Importantly, NET-bound NE retains enzymatic activity, resulting in more specific and sustained damaging effects compared to free NE [97,98]. Neutrophils generate reactive oxygen species (ROS) specifically through the NADPH oxidase complex. Hyperglycemia amplifies ROS production by enhancing NADPH oxidase activity. ROS not only directly damage cells but also promote NET formation by activating PAD4, establishing an “oxidative stress-NETs” positive feedback loop [99].

The MPO system represents the most characteristic and potent oxidative damage mechanism of neutrophils [100]. MPO is the most abundant peroxidase in neutrophil azurophilic granules, accounting for approximately 5% of total neutrophil protein. During neutrophil activation, MPO is released into the extracellular space or into phagolysosomes, where it catalyzes the generation of highly toxic hypochlorous acid (HOCl) using hydrogen peroxide (H_2_O_2_) and chloride ions (Cl^−^). HOCl is a strong oxidant that irreversibly oxidizes cysteine and methionine residues in proteins, leading to conformational changes and functional loss. It also induces lipid peroxidation of cell membranes, disrupting membrane integrity, and causes DNA strand breaks and base modifications, triggering apoptosis or necrosis [101]. MPO-derived HOCl directly damages hepatocytes and hepatic sinusoidal endothelial cells, serving as a critical effector molecule for tissue necrosis. Emerging findings offer the first definitive evidence that HOCl mediates post-translational modification of proteins in NETs, especially histone H4 [102], revealing a critical mechanistic link to sterile inflammatory diseases such as hepatic ischemia–reperfusion injury. Additionally, MPO further exacerbates hepatic inflammation and fibrosis by oxidatively modifying low-density lipoproteins and activating matrix metalloproteinases [103].

## 5. Neutrophil Intercellular Interaction Network in Hepatic Ischemia–Reperfusion Injury

**Figure 5:**
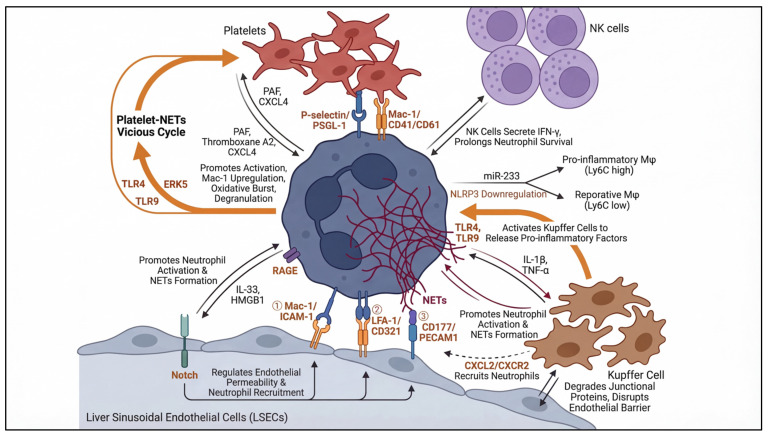
Neutrophil-centered intercellular crosstalk networks in hepatic ischemia–reperfusion injury (HIRI). HIRI is a critical perioperative complication of liver transplantation and major hepatectomy, and neutrophil-centered intercellular crosstalk is the core driver of sterile inflammatory burst and tissue damage in HIRI. This schematic illustrates the neutrophil-centered multi-cellular interaction networks in the hepatic sinusoid microenvironment, including: (1) Neutrophil–platelet crosstalk, mediated by adhesion molecules, forms a NETs-related cross-activation vicious cycle to aggravate microcirculation disturbance; (2) Neutrophil–liver sinusoidal endothelial cell (LSEC) crosstalk, which regulates neutrophil recruitment, adhesion and transendothelial migration via multiple molecular pairs, and forms a secondary damage cycle; (3) Neutrophil–Kupffer cell crosstalk, which forms a pro-inflammatory amplification loop, while neutrophils exert bidirectional regulation on macrophage polarization; (4) Neutrophil–natural killer (NK) cell crosstalk, which participates in inflammatory regulation in HIRI.

Neutrophils do not act in isolation during HIRI. Instead, they engage in dynamic bidirectional interactions with multiple cell types in the liver microenvironment, including platelets, LSECs, and macrophages. These intercellular communications form a complex regulatory network that coordinates the initiation, amplification, and resolution of the inflammatory response. Dysregulation of these interactions drives the transition from physiological defense to pathological tissue injury and represents promising but challenging therapeutic targets (Figure 5).

### 5.1. Neutrophil–Platelet Interactions

HIRI activates both platelets and neutrophils almost simultaneously, and their direct physical interaction is the primary trigger for rapid NET formation in early reperfusion and the initiating event of the inflammation–thrombosis cycle. Notably, platelet transfusion has been shown to exert pro-inflammatory effects on neutrophil function, particularly in the context of cirrhosis. Activated platelets bind to PSGL-1 on the neutrophil surface via surface P-selectin. This interaction exerts two synergistic pro-inflammatory effects: first, it directly activates the Syk-Ca^2+^-PAD4 pathway, triggering rapid vital NETosis. Notably, under conditions of altered hepatic sinusoidal shear stress, the potential role of mechanosensitive ion channels in this process warrants further investigation. Second, it upregulates the expression and affinity of Mac-1 on the neutrophil surface, enhancing their adhesive capacity and transendothelial migration potential. Furthermore, activated platelets release soluble mediators, including platelet-activating factor (PAF) and CXCL4, which directly induce neutrophil respiratory burst and degranulation, further amplifying their pathogenic effector functions [104].

Recent studies have uncovered a self-sustaining cross-activation loop between platelets and NETs. In models of bacterial sepsis and lung injury, platelet TLR4 activation promotes NET formation through the ERK5 pathway. Conversely, DNA released within NETs activates platelet TLR9, further enhancing platelet activation and aggregation [105,106]. NETs also provide a physical scaffold for platelet aggregation and coagulation factor activation, accelerating microthrombus formation within the hepatic sinusoids. This establishes a vicious cycle of “platelet activation → NET release → further platelet activation”, driving progressive microcirculatory dysfunction.

Preclinical studies consistently demonstrate that pharmacological blockade of P-selectin function disrupts neutrophil–platelet aggregates, reduces NET formation, and alleviates liver injury in mouse models of HIRI, providing a rationale for upstream therapeutic intervention [107,108]. However, clinical translation of this strategy faces a fundamental challenge: P-selectin antibodies not only inhibit pathological inflammation but also impair physiological hemostasis, significantly increasing the risk of perioperative bleeding, which remains a major limitation for their clinical application.

### 5.2. Neutrophil–Endothelial Cell Interactions

LSECs are not merely passive physical barriers but rather active regulators of neutrophil recruitment and activation. The dynamic interplay between neutrophils and LSECs constitutes the core of the leukocyte adhesion cascade. Emerging evidence has identified Notch signaling, a pathway traditionally associated with cell differentiation and fate determination, as a critical regulator of endothelial-mediated inflammatory responses in HIRI [109]. Endothelial Notch signaling actively modulates neutrophil recruitment by regulating the expression of endothelial adhesion molecules such as endomucin (EMCN) and modulating chemokine secretion, thereby altering endothelial barrier permeability to neutrophils. This indicates that LSECs do not simply upregulate adhesion molecules in response to inflammatory stimuli but rather actively function as gatekeepers of leukocyte entry into tissue through intrinsic signaling pathways. Indeed, blockade of lymphocyte function-associated antigen-1 (LFA-1), which mediates leukocyte adhesion via binding to EMCN, abrogates the exacerbation of HIRI induced by Notch activation [1,110].

In a mouse model of warm hepatic ischemia–reperfusion injury (1 h ischemia followed by 6 h reperfusion), increased LSEC activation promotes the release of interleukin-33 (IL-33), which in turn drives neutrophil activation and NET formation [13]. CD321 (junctional adhesion molecule-A, JAM-A) plays a critical role in amplifying neutrophil–endothelial cell interactions: TNF-α induces CD321 redistribution on the endothelial cell surface, enhancing its binding affinity for neutrophil LFA-1 and promoting neutrophil activation and release of damage mediators. Anti-CD321 antibodies block this interaction, reducing endothelial barrier disruption through a mechanism independent of the Mac-1 pathway, representing a novel target for combination therapy [10].

Furthermore, HMGB1 released from damaged LSECs activates neutrophils via the RAGE receptor, promoting NET formation. In turn, NETs degrade endothelial intercellular junction proteins, further compromising vascular barrier integrity. Notably, CD177^+^ neutrophils exhibit enhanced transendothelial migration capacity through specific interaction with endothelial platelet endothelial cell adhesion molecule-1 (PECAM-1) [111], highlighting the functional heterogeneity of neutrophil subsets in their interactions with endothelial cells. In addition to CD177^+^ neutrophils and aged neutrophils, recent single-cell RNA sequencing studies have identified Ly6G^+^CD11b^+^CXCR4^+^ pro-inflammatory neutrophil subsets and Ly6G^+^CD11b^+^CD206^+^ reparative neutrophil subsets in HIRI models.

### 5.3. Neutrophil–Macrophage Interactions

Kupffer cells, the resident macrophages of the liver, are among the first cells to be activated during HIRI, releasing large amounts of inflammatory mediators that initiate and amplify the inflammatory response. NET components, including HMGB1 and extracellular DNA, activate Kupffer cells via TLR9 and TLR4 receptors, promoting the secretion of TNF-α, IL-1β, and other pro-inflammatory cytokines, which further drive neutrophil recruitment and activation [112]. Importantly, Kupffer cells have a limited capacity to phagocytose and clear NETs, and impaired NET clearance leads to sustained inflammation and progressive tissue injury. Single-cell RNA sequencing studies have revealed significant functional heterogeneity of hepatic macrophages in HIRI. Specifically, Cxcl2^+^ macrophages exhibit a senescence-associated secretory phenotype (SASP) and recruit neutrophils to the aged liver via the CXCL2-CXCR2 axis. These macrophages also stimulate NET formation by secreting IL-1β and TNF-α. Targeting the CXCL2-CXCR2 axis limits neutrophil migration to the liver and alleviates age-related exacerbation of HIRI [113].

Importantly, neutrophils also exert beneficial immunomodulatory effects by promoting the conversion of macrophages from a pro-inflammatory (*Ly6C*^high^ *CX3CR1*^low^) phenotype to a reparative (*Ly6C*^low^ *CX3CR1*^high^) phenotype, a mechanism well documented in models of acute liver injury [114,115]. Recent studies have identified neutrophil-derived microRNA-233 (miR-233) as a key mediator of this process, which downregulates NLRP3 inflammasome expression in macrophages [116]. Both miR-233 knockout mice and neutrophil-depleted mice exhibit persistent accumulation of pro-inflammatory macrophages, accompanied by enhanced liver inflammation and fibrosis [117]. Mechanistically, PP4 orchestrates macrophage-neutrophil crosstalk under septic conditions; its deficiency triggers aberrant CCL5/CCR5 signaling and excessive neutrophil activation, highlighting a novel actionable target for fine-tuning innate immune responses in HIRI [118]. Furthermore, a charge-adaptive neutrophil-targeting nanoparticle system (CPS@BA) capable of simultaneously scavenging and inhibiting NET formation disrupts the detrimental NET-macrophage crosstalk by blocking the CXCL3-CXCR2 axis, exhibiting broad therapeutic potential in all cell-free DNA-driven inflammatory diseases, including HIRI [119].

### 5.4. Neutrophil–NK Cells Interactions

In addition to macrophages, neutrophils also interact with natural killer (NK) cells during HIRI. Neutrophil–NK cell crosstalk promotes increased interferon-γ (IFN-γ) secretion by NK cells while prolonging neutrophil survival [114], further modulating the inflammatory microenvironment.

## 6. Functional Duality of Neutrophils and Inflammation Resolution

Although the detrimental role of neutrophils in HIRI has been extensively documented, accumulating evidence in recent years has revealed their remarkable functional duality: in the late stage of inflammation, neutrophils can undergo phenotypic polarization toward an anti-inflammatory and reparative phenotype, participating in inflammation resolution and tissue regeneration. This property provides new perspectives for the development of precision therapeutic interventions.

The significant functional plasticity of neutrophils is primarily manifested as bidirectional polarization toward N1 and N2 phenotypes. The N1 phenotype represents the classical pro-inflammatory and tissue-damaging subset, characterized by high expression of ROS, MPO, NE, and NET-associated molecules, and exacerbates liver tissue injury through the release of pro-inflammatory cytokines and cytotoxic mediators. In contrast, the N2 phenotype is an anti-inflammatory and reparative subset with enhanced phagocytic capacity and high expression of repair factors such as IL-10, TGF-β, and HGF, which clear necrotic debris and suppress excessive inflammatory responses [120]. Although direct and specific markers for the N2 phenotype in HIRI have not been fully elucidated, cross-disease evidence demonstrates that a dynamic transition from “early N1-dominant injury” to “late N2-dominant repair” exists in both myocardial and cerebral ischemia models, and the pathological progression of HIRI follows a similar pattern [121,122]. Deng et al. confirmed that in a mouse liver injury model, neutrophils can secrete HGF to promote hepatocyte proliferation, a reparative function that is highly consistent with the characteristics of the N2 phenotype [123].

This property suggests that HIRI treatment should avoid non-specific suppression of neutrophils and instead adopt a “precise regulation” strategy: inhibiting N1 polarization and NET formation in the early phase to attenuate injury, and promoting N2 transition in the mid-phase via signals such as TGF-β and interleukin-4 (IL-4) or interventions like mesenchymal stem cell-derived extracellular vesicles (MSC-EVs). This approach preserves their antimicrobial defense function while harnessing their reparative properties to accelerate hepatic tissue regeneration. Future studies are required to identify specific markers and regulatory molecules of the N2 phenotype in HIRI, which will provide a theoretical foundation for achieving spatiotemporally precise reprogramming of neutrophil function.

## 7. Therapeutic Strategies and Clinical Translation Challenges

Based on the neutrophil-mediated pathogenic mechanisms described above, multiple classes of targeted therapeutic strategies have been developed, all of which have shown significant liver protection in preclinical animal models. However, to date, no neutrophil-targeted drug has been approved for the clinical treatment of HIRI. The core reasons for this translational failure lie in three fundamental challenges: the difficulty of balancing therapeutic efficacy and safety, the translational gap between animal models and clinical reality, and the limited efficacy of single-target therapies. Following the temporal logic of upstream recruitment blockade, midstream signal inhibition, and downstream effector neutralization, this chapter systematically reviews the research progress, clinical translation bottlenecks, and optimization directions of core therapeutic strategies (Table 1).

### 7.1. Targeting Neutrophil Recruitment

#### 7.1.1. Inhibition of Adhesion Molecules

As the core member of the β2 integrin family, Mac-1 (CD11b/CD18) mediates firm adhesion of neutrophils to endothelial cells and subsequent transendothelial migration, making it a theoretically ideal therapeutic target. Preclinical studies have consistently demonstrated that functional inactivation of neutrophils using anti-Mac-1 or anti-CD18 monoclonal antibodies significantly attenuates HIRI [124]. Beyond direct blockade, modulating Mac-1 expression through alternative approaches represents a promising direction. For example, hypertonic saline pretreatment has been shown to attenuate HIRI by inhibiting Mac-1 expression on neutrophils and ICAM-1 expression in the liver [125]. Given that hypertonic saline is already widely used in clinical perioperative settings, this strategy holds considerable translational potential. However, clinical translation of this strategy has faced formidable challenges.

Two core unresolved controversies directly restrict the clinical translation of Mac-1-targeted therapies:

- Organ-specific efficacy contradiction: While Mac-1 inhibition consistently alleviates HIRI-induced hepatic necrosis in preclinical models, multiple independent studies have verified that Mac-1 blockade does not reduce neutrophil sequestration in the liver parenchyma, and only attenuates distant organ injury [35,36]. This discrepancy cannot be explained by the classical leukocyte adhesion cascade, as the unique sinusoidal microcirculation of the liver may limit the efficacy of adhesion molecule blockade alone [33]. The non-classical regulatory mechanisms of Mac-1 in the low-shear, fenestrated hepatic sinusoidal microenvironment remain incompletely elucidated, which prevents us from fully explaining why the effects of Mac-1 blockade differ between the liver and other organs, and represents a key unresolved issue for future research [126].
- Preclinical efficacy and clinical translation paradox: Despite encouraging preclinical hepatoprotective effects of Mac-1-targeted agents (including anti-Mac-1 monoclonal antibodies and recombinant neutrophil inhibitory factor, rNIF) in HIRI animal models, the clinical translation of Mac-1-targeted therapy has encountered major setbacks [127]. rNIF, which specifically targets the Mac-1 I-domain, showed promising efficacy in animal models but failed to improve patient outcomes in the phase II clinical trial for ischemic stroke, leading to the termination of its subsequent development [127]. The core barrier to clinical translation is the efficacy-safety balance dilemma: Mac-1 is indispensable for neutrophil-mediated anti-pathogen host defense, and systemic blockade of Mac-1 significantly increases the risk of severe perioperative infections, which is particularly fatal for patients undergoing liver transplantation or major hepatectomy, as any potential therapeutic benefit would be directly offset by the increased infection risk [128]. Notably, the ligand-specific Mac-1 blockade strategy that separates pathological inflammation and physiological host defense has not been validated in HIRI preclinical models to date.

#### 7.1.2. Blockade of Chemotactic Signals

Chemokines such as CXCL1, CXCL2, and CXCL8 play a central role in neutrophil recruitment via their receptors CXCR1 and CXCR2. Small molecule inhibitors such as reparixin, a non-competitive allosteric inhibitor of CXCR1/2, have been shown to effectively reduce neutrophil infiltration in the liver and lungs, decrease serum ALT levels, and attenuate histological damage in HIRI models. However, its phase II trial for renal transplantation IRI has been discontinued [19]. Genetic studies further support this target: CXCR2 knockout mice exhibit significantly reduced liver injury markers following HIRI compared to wild-type mice [129].

The high degree of redundancy in the chemokine system, where multiple pathways can drive neutrophil recruitment, is the primary reason for the limited efficacy of single-agent chemokine blockade [130]. Therefore, combination therapy targeting multiple chemokine axes represents the major future direction for this strategy.

### 7.2. Midstream Signaling Inhibition Strategies

Midstream signaling inhibition strategies aim to suppress neutrophil activation and effector functions, with a therapeutic window of early reperfusion. The core target is Syk kinase, while inhibitors of the MAPK, PI3K, and NF-κB pathways are also under investigation.

#### 7.2.1. Syk Inhibitors

As the critical downstream kinase of immune receptors and the core hub of the Mac-1 signaling axis, Syk inhibition effectively suppresses NETosis and the amplification of sterile inflammatory cascades, with well-documented hepatoprotective effects in multiple HIRI preclinical models [48]. Notably, several Syk inhibitors have been approved for clinical use in hematological malignancies, providing a mature foundation for drug repurposing in HIRI. Among them, ruxolitinib, a JAK1/2 inhibitor with off-target Syk inhibitory activity, has been formally approved for the treatment of myelofibrosis [131]; a small-scale clinical study further showed that ruxolitinib can reduce postoperative inflammatory responses in liver transplant recipients, albeit with a slightly increased risk of perioperative infection.

Despite these promising preclinical findings and drug repurposing potential, no Syk inhibitor has entered prospective clinical trials for HIRI to date [132]. The core barrier to clinical translation lies in the non-specific expression pattern of Syk: it is not exclusively expressed in neutrophils, but ubiquitously distributed in multiple innate and adaptive immune cell populations, including B cells and macrophages. Consequently, systemic Syk inhibition inevitably induces broad immunosuppression, significantly increasing the risk of perioperative bacterial and fungal infections, which is a life-threatening adverse event for patients undergoing liver transplantation or major hepatectomy. To address this fundamental challenge, generation of neutrophil-specific Syk conditional knockout mice is an urgent and unmet need to clearly define the cell-autonomous role of Syk in HIRI pathogenesis, and to distinguish the on-target therapeutic effect from off-target immunosuppressive effects.

#### 7.2.2. PI3K/Akt Inhibitors

The phosphoinositide 3-kinase pathway is a core regulator of neutrophil migration and activation, and PI3K inhibitors exhibit significant anti-inflammatory effects by modulating neutrophil chemotactic behavior and effector functions [65]. The development of isoform-selective inhibitors (particularly PI3Kγ/δ) that preferentially target immune cells holds great promise for reducing systemic off-target effects. Notably, PI3Kγ/δ isoform-selective inhibitors are already under clinical investigation in oncology and immunology, providing a mature foundation for drug repurposing in HIRI [133]. Since PI3Kδ and PI3Kγ are primarily expressed in immune cells with very low basal expression in hepatocytes, p110γ-specific inhibitors have the potential to be repurposed from “targeted therapy” to “immuno-selective therapy” for HIRI. Several preclinical studies have confirmed that PI3Kγ inhibitors significantly reduce HIRI in mice without obvious hepatotoxicity, showing favorable translational potential [134,135].

Two core unresolved controversies directly restrict the clinical translation of PI3K/Akt-targeted therapies:

- Cell-specific functional paradox: The role of the PI3K/Akt pathway in HIRI remains highly contradictory across preclinical studies. On the one hand, numerous studies indicate that activation of the PI3K/Akt pathway in hepatocytes exerts strong hepatoprotective effects by inhibiting oxidative stress and apoptosis [75]; consistent with this, administration of the non-selective PI3K inhibitor PX866 leads to exacerbated hepatic IR injury in mice [65]. On the other hand, in neutrophils, activation of the PI3K/Akt pathway is a key driver of migration, activation, and NET formation, and inhibition of this pathway significantly alleviates inflammatory liver injury [63]. This starkly opposite cell-specific effect makes systemic non-selective PI3K inhibition clinically infeasible, and represents the most fundamental scientific challenge in targeting this pathway.
- Isoform heterogeneity-induced efficacy contradiction: Different PI3K isoforms play highly heterogeneous roles in neutrophil activation and NET formation: PI3Kα and PI3Kγ are involved in NET formation induced by various stimuli, while the roles of other isoforms are highly stimulus-dependent [63]. Most early preclinical studies used non-selective pan-PI3K inhibitors, leading to highly contradictory efficacy and safety outcomes across studies. Although isoform-selective PI3Kγ/δ inhibitors show promising preclinical efficacy, their specific regulatory mechanisms, long-term in vivo safety, and optimal dosing regimen in the HIRI perioperative setting remain to be fully validated in well-designed clinical studies.

#### 7.2.3. MAPK Inhibitors

The mitogen-activated protein kinase family, especially p38 MAPK, is crucial for inflammatory signal transduction. p38 inhibitors have shown potential in reducing neutrophil infiltration and inhibiting their function in various inflammatory models [50]. A key limitation of current studies is the cell-specific bidirectional effect of the MAPK pathway: while inhibiting p38/JNK in neutrophils alleviates inflammatory injury, inhibiting the same pathway in hepatocytes may impair their intrinsic anti-apoptotic capacity. This fundamental paradox represents the core translational bottleneck for MAPK inhibitors. Future studies should use neutrophil-specific promoters (such as MRP8) to drive Cre recombinase expression for conditional knockout of pathway components, thereby elucidating their cell-specific contributions [136]. Moreover, most studies have focused on individual MAPK subfamilies in isolation, with limited investigation of crosstalk and compensatory mechanisms among the three branches, which may explain the limited efficacy of single-target inhibitors [137].

A common limitation of all kinase inhibitors is their broad expression across multiple cell types, leading to systemic off-target effects and toxicity. Therefore, identifying neutrophil-enriched signaling nodes or developing targeted delivery technologies is crucial. The core future directions are neutrophil-specific targeted delivery and isoform-selective inhibitors to resolve cell-specific functional paradoxes and reduce systemic toxicity. Bruton’s tyrosine kinase (Btk), expressed in B cells and myeloid cells including neutrophils, has emerged as an attractive new target. Btk inhibitors interfere with integrin (including Mac-1) signaling, thereby inhibiting neutrophil recruitment and activation [138], offering a new direction for exploration in HIRI therapy.

### 7.3. Downstream Effector Neutralization Strategies

Downstream strategies target the pathogenic effector molecules released by activated neutrophils, offering a wider therapeutic window that extends into the mid-reperfusion phase.

#### 7.3.1. Inhibition of NET Formation

Peptidylarginine deiminase 4 (PAD4) is the critical executioner enzyme for NETosis, and inhibiting its activity directly prevents chromatin decondensation. Multiple small molecule PAD4 inhibitors have been developed, including Cl-amidine, GSK484 and its derivatives, and the novel specific inhibitor YW4-03 [139,140]. In various HIRI animal models, both prophylactic and therapeutic administration of PAD4 inhibitors significantly reduce NET formation in liver tissue, decrease serum transaminase levels, attenuate hepatic inflammatory infiltration and necrosis, and improve microcirculatory perfusion [141]. These studies strongly validate PAD4 as an effective therapeutic target for HIRI. Furthermore, recent studies have shown that PAD4 inhibitors combined with sub-inhibitory concentrations of DNase I synergistically disrupt NETs and reduce histone release.

Despite encouraging preclinical data, no clinical trials of PAD4 inhibitors specifically for HIRI have been publicly reported. Their safety, effective dose, and optimal administration timing in humans remain to be further explored. To achieve precise drug accumulation in the injured liver, researchers are exploring nanotechnology-based targeted delivery systems. Studies have attempted to encapsulate PAD4 inhibitors such as Cl-amidine in liposomes or polymeric nanoparticles. For example, a designed liposomal system enables responsive drug release under the stimulation of the inflammatory microenvironment, achieving targeted therapy at the lesion site [142]. Another study demonstrated that nanoparticle-delivered PAD4 inhibitors effectively accumulate at the lesion site and sustainably inhibit NET formation in an arterial injury model [143]. These innovative delivery strategies hold great promise for significantly improving the efficacy of PAD4 inhibitors in HIRI treatment while reducing potential side effects.

#### 7.3.2. Degradation of Preformed NETs (DNase I)

DNase I is a non-specific endonuclease that cleaves DNA strands, making it the most direct and effective tool for degrading NETs. Recombinant human DNase I (rhDNase, trade name Pulmozyme^®^) disrupts the DNA backbone of NETs, rapidly dismantling their web-like structure and releasing sequestered histones, MPO, and other toxic proteins for dilution in the circulation and clearance by hepatic phagocytes such as Kupffer cells [96].

Exogenous administration of DNase I has demonstrated significant protective effects in ischemia–reperfusion injury models of multiple organs, including the heart, kidney, intestine, and liver. In mouse HIRI models, intravenous DNase I effectively reduces circulating NET markers such as MPO-DNA complexes, confirming its in vivo efficacy in NET degradation [38]. Importantly, DNase I treatment significantly attenuates liver tissue damage, as evidenced by decreased serum ALT/AST levels and improved liver pathology scores [144].

rhDNase I is not a novel drug; it has long been approved for the treatment of cystic fibrosis (CF) to thin airway secretions by degrading DNA released from dead neutrophils. It has demonstrated good safety and tolerability in clinical trials for CF [145]. Phase II clinical trials are currently ongoing for acute pancreatitis and acute respiratory distress syndrome [146]. However, no phase I or II clinical trials of DNase I specifically for perioperative HIRI in liver transplant patients have been completed and published with detailed results. These potential risks require careful evaluation in rigorous clinical trials.

A theoretical concern is that rapid degradation of large amounts of NETs may release high concentrations of histones and other toxic components in a short period, potentially exacerbating local cellular injury. Sequential combination therapy with anti-histone antibodies is a key strategy to address this issue. Additionally, some studies have suggested that DNase I administration may be associated with increased inflammation or thrombosis risk under certain specific conditions [147].

#### 7.3.3. Neutralization of NET Toxic Components

- Targeting histones: In mouse HIRI models, pre-administration of neutralizing monoclonal antibodies against histone H3 or H4 significantly attenuates liver injury. These antibodies bind and neutralize circulating histones, preventing their direct cytotoxic effects on hepatocytes and activation of inflammatory pathways, resulting in reduced serum ALT levels, smaller necrotic areas, and downregulated expression of pro-inflammatory cytokines such as TNF-α and IL-6 [147]. Beyond direct neutralization, modulating histone modifications represents an alternative approach. For example, the novel histone deacetylase (HDAC) inhibitor LP342 has been shown to protect against HIRI in mice by increasing histone acetylation levels, thereby attenuating oxidative stress and mitochondrial dysfunction [148].
- Targeting neutrophil elastase (NE): NE is a key protease that degrades the extracellular matrix and amplifies inflammation. The specific inhibitor sivelestat sodium has been shown to dose-dependently inhibit neutrophil migration to the vascular wall and significantly attenuate HIRI [149]. It also demonstrates broad protective effects in ischemia–reperfusion models of the heart, kidney, and other organs [150,151,152]. However, concerns remain that long-term or systemic inhibition of NE and MPO may increase infection risk due to their important roles in host defense. Therefore, developing local administration or targeted delivery systems to reduce systemic exposure is a critical future direction. A notable study constructed “NE-based nanovesicles” that specifically target and accumulate at inflammatory injury sites, enabling efficient drug delivery and significantly enhancing protection against ischemia–reperfusion injury [153].
- Targeting MPO: MPO is a key component of NETs and a marker of neutrophil activation. Theoretically, blocking its enzymatic activity using anti-MPO antibodies or small molecule inhibitors could attenuate oxidative stress damage. However, similar to anti-histone antibodies, no clinical trials of anti-MPO antibodies in liver transplant patients have been conducted to date.
- Targeting HMGB1: Studies have demonstrated that neutralizing anti-HMGB1 antibodies significantly attenuate liver injury in HIRI animal models, confirming HMGB1 as an effective therapeutic target in HIRI [70,154].

### 7.4. Nano-Targeted Delivery Systems and Extracellular Vesicles for Neutrophil-Targeted HIRI Therapy

With the deepening understanding of neutrophil heterogeneity and functional duality in HIRI, nano-targeted delivery systems and extracellular vesicles (EVs) have emerged as promising tools to achieve precise regulation of neutrophil function, addressing the limitations of traditional systemic therapies such as off-target effects and insufficient tissue penetration.

Nano-targeted delivery systems leverage the unique biological characteristics of neutrophils for active or passive targeting [155]. For instance, neutrophil membrane-camouflaged nanoparticles inherit surface antigens like CXCR2 and Mac-1, enabling specific accumulation in injured liver tissue and controlled release of PAD4 inhibitors, DNase I or anti-inflammatory drugs in response to ROS or pH signals. Notably, a breakthrough charge-adaptive nanoparticle (CPS@BA) has recently addressed the long-standing cytotoxicity limitation of cationic NET-capturing materials. This system is constructed by conjugating sialic acid (SA) to carboxymethyl chitosan-polyethyleneimine (CMCS-PEI) copolymer and encapsulating the calcium chelator BAPTA-AM (BA). It dynamically reverses its surface charge from negative to positive upon sensing the acidic microenvironment around activated neutrophils, enabling rapid capture of negatively charged DNA-based NETs; when pH returns to neutral after inflammation resolution, it switches back to biocompatible negative charge to minimize systemic toxicity. Meanwhile, charge adaptation triggers size adaptation and controlled BA release, which inhibits further NET formation by chelating intracellular Ca^2+^ and blocking PAD4 activation [119]. This dual strategy of simultaneous NET clearance and inhibition effectively disrupts the detrimental crosstalk between NETs and macrophages via the CXCL3-CXCR2 axis, showing broad therapeutic potential for all cell-free DNA-driven inflammatory diseases including HIRI. A novel neutrophil-targeted, ROS- and pH-responsive nanoplatform co-delivering dioscin and siICAM-1 can efficiently accumulate in infarcted myocardium, suppress neutrophil infiltration and inflammatory damage, ameliorate mitochondrial dysfunction, and effectively restore cardiac function after myocardial infarction, representing a promising and biosafe spatiotemporal therapeutic strategy with high clinical translation potential [156].

EVs, especially mesenchymal stem cell-derived EVs (MSC-EVs) and neutrophil-derived EVs, exhibit inherent biocompatibility and natural targeting capabilities [157]. MSC-EVs repair neutrophil mitochondrial dysfunction and suppress NET formation through the “mitochondrial rescue” mechanism by transferring functional mitochondria and microRNAs. Engineered neutrophil-derived EVs loaded with anti-inflammatory cytokines or small-molecule inhibitors can specifically block pro-inflammatory signaling cascades such as Mac-1-Syk-NF-κB in activated hepatic neutrophils. ROS-responsive EV systems further enable precise payload release in the high-oxidative-stress microenvironment of HIRI, simultaneously alleviating oxidative stress and promoting neutrophil phenotypic switching toward N2 [158].

Despite these advances, clinical translation still faces challenges including standardized large-scale production of EVs, precise control of drug loading and release kinetics, and long-term safety evaluation of nanomaterials in vivo.

### 7.5. Core Clinical Translational Bottlenecks of Neutrophil-Targeted Therapies for HIRI

Neutrophil-targeted therapeutic strategies have shown favorable hepatoprotective efficacy in multiple preclinical HIRI models, but their clinical translation is hindered by multiple systematic and interconnected barriers. This section systematically integrates and elaborates on the universal core translational bottlenecks of neutrophil-targeted HIRI therapies, classified into four key dimensions for in-depth analysis.

#### 7.5.1. Systemic Immunosuppression and Perioperative Infection Risk

The most fatal and prevalent translational barrier shared by nearly all neutrophil-targeted interventions is the imbalance between therapeutic efficacy and the maintenance of innate antimicrobial host defense, which severely limits the clinically applicable dose and further narrows the already narrow therapeutic window. This risk is the primary reason why most neutrophil-targeted agents have not advanced into clinical trials for HIRI, as the majority of therapies are terminated at the preclinical stage due to fatal infection-related adverse events observed in clinical trials of other inflammatory diseases. Representative clinical verification cases have fully confirmed this risk: recombinant neutrophil inhibitory factor (rNIF) targeting Mac-1 was discontinued in phase II clinical trials for ischemic stroke due to severe infection adverse events [127], and both Syk inhibitors and CXCR2 inhibitors showed a significantly increased incidence of severe infections in clinical studies of chronic inflammatory diseases [128,132]. To address this barrier, the core solution directions include the development of neutrophil-specific targeted delivery systems, ligand-specific intervention strategies that separate pathological inflammation from physiological host defense, and short-term pulsed administration in the early reperfusion stage to reduce the risk of long-term immune suppression.

#### 7.5.2. Target Non-Specificity and Cell-Specific Functional Paradox

The second core systematic bottleneck is the non-specific expression pattern of target molecules and the cell-specific functional paradox, which leads to inevitable off-target toxicity and even the exacerbation of liver injury. Systemic non-selective inhibition of most core targets causes unavoidable off-target adverse events, including the exacerbation of hepatocyte apoptosis and impairment of liver regeneration, which completely offsets the expected therapeutic efficacy and renders most pan-inhibitors clinically infeasible [75]. Representative preclinical and clinical cases have verified this challenge: the non-selective pan-PI3K inhibitor PX866 significantly exacerbated hepatic IR injury in mouse models due to the inhibition of hepatoprotective PI3K signaling in hepatocytes [65], and systemic Syk inhibitors caused severe myelosuppression and broad immunosuppression in clinical trials of hematological and inflammatory diseases [132]. The targeted solution directions for this bottleneck include the development of isoform-selective inhibitors targeting immune cell-specific subtypes (e.g., PI3Kγ/δ inhibitors), neutrophil-specific gene manipulation to verify the cell-autonomous function of core targets, and biomimetic targeted delivery systems to reduce off-target drug distribution in hepatic parenchymal cells.

#### 7.5.3. Limitations of Preclinical Animal Models and Translational Gap

The fundamental bottleneck leading to the frequent “effective in mice but ineffective in humans” phenomenon in clinical translation is the huge gap between conventional preclinical animal models and real clinical practice, which results in serious overestimation of preclinical efficacy and means that the results of most preclinical studies cannot guide the rational design of clinical trials [159]. This translational gap stems from three core discrepancies: first, the huge difference in baseline characteristics between healthy young inbred mice used in most preclinical studies and clinical patients with comorbidities such as advanced age, liver steatosis, cirrhosis, and diabetes [160]; second, the critical difference in administration timing between prophylactic intervention in preclinical studies and therapeutic intervention only after reperfusion in real clinical practice; third, the inherent species differences in neutrophil biology between mice and humans, including the expression pattern of chemokine receptors and the regulatory mechanism of NET formation. Almost all neutrophil-targeted agents that show efficacy in mouse HIRI models have failed to achieve the expected efficacy in subsequent clinical studies of ischemic diseases, and species differences in chemokine receptor expression have led to the failure of multiple murine-specific agents in human trials [161]. To break this translational gap, the core solution directions include the establishment of clinically relevant HIRI models (e.g., aged, liver steatosis, cirrhosis, and diabetes comorbidity models), standardized administration timing completely consistent with clinical practice, and humanized neutrophil mouse models to reduce species differences.

#### 7.5.4. Uncertainty of the Optimal Therapeutic Time Window

HIRI is a highly dynamic and strictly time-dependent pathological process, and the uncertainty of the optimal therapeutic time window for different intervention strategies directly leads to unreasonable clinical trial design, incorrect intervention timing, and failure to achieve expected therapeutic efficacy, which greatly reduces the clinical application value of effective preclinical strategies [162]. The core of this bottleneck is that different neutrophil-targeted strategies have completely different optimal intervention windows along the time course of HIRI: upstream recruitment blockade strategies have an extremely narrow effective window only within 1 h after reperfusion, and delayed administration beyond this window shows almost no efficacy, as verified in preclinical studies. Consistent with this, most clinical trials of anti-adhesion molecule therapies for ischemic diseases used delayed intervention beyond the effective window, resulting in no significant therapeutic efficacy [163]. The core solution directions to address this uncertainty include preclinical studies to clearly define the strategy-specific optimal therapeutic window, the development of biomarkers for dynamic monitoring of neutrophil activity and NET levels to guide personalized intervention timing, and the formulation of staging intervention strategies according to the time course of HIRI. Collectively, the four core translational bottlenecks mentioned above are interrelated and mutually reinforcing, and breaking through these barriers is the core prerequisite for the clinical translation of neutrophil-targeted therapies for HIRI.

**Table 1:** Therapeutic strategies, research progress and translational challenges of targeting neutrophils against HIRI.

Compound	Clinical Stage	Representative Agents	Critical Safety Profile	Ref.
Anti-Mac-1 (CD11b) monoclonal antibody	Preclinical	Anti-CD11b monoclonal antibody, M1/70 antibody	Dose-dependent impairment of neutrophil antimicrobial host defense; increased risk of systemic bacterial/fungal infections with systemic administration	[124]
Anti-CD18 monoclonal antibody	Preclinical	Anti-CD18 monoclonal antibody, IB4 antibody	Systemic blockade significantly increases perioperative severe infection risk; impaired innate immune defense against pathogens	[124]
Recombinant neutrophil inhibitory factor (rNIF)	Phase II (Discontinued for ischemic stroke; Preclinical for HIRI)	Recombinant neutrophil inhibitory factor (rNIF)	Severe infection-related adverse events reported in phase II clinical trials; dose-dependent systemic immunosuppression	[124]
Hypertonic saline	Marketed (Widely used in clinical perioperative settings; Preclinical for HIRI-specific indication)	3% Hypertonic saline	Excellent safety profile in standard clinical perioperative use; no increased infection risk reported at conventional therapeutic doses	[125]
Reparixin (CXCR1/2 inhibitor)	Phase II (Discontinued for renal IRI; Preclinical for HIRI)	Reparixin (DF1681B)	Mild and reversible hematological adverse events; low but non-negligible infection risk with long-term continuous administration	[19]
CXCR2 knockout (genetic)	Preclinical (Genetic mouse model only; no clinical development)	CXCR2 global/conditional knockout mice	No clinical safety data available; theoretical risk of impaired antimicrobial host defense with systemic CXCR2 inhibition	[129]
Syk inhibitors (e.g., ruxolitinib)	Marketed (for myelofibrosis; Preclinical for HIRI; Small clinical study in liver transplantation)	Ruxolitinib, Fostamatinib	Broad systemic immunosuppression; significantly increased risk of perioperative bacterial/fungal infections; reversible myelosuppression, thrombocytopenia and anemia in clinical use	[48,131]
PI3K inhibitors (isoform-selective, e.g., PI3Kγ/δ)	Preclinical for HIRI; Phase II for other inflammatory diseases	IPI-549 (PI3Kγ), Duvelisib (PI3Kδ/γ), CAL-101	Favorable safety profile in preclinical HIRI studies; minimal off-target hepatotoxicity due to immune cell-specific isoform targeting; low infection risk in early clinical trials of other inflammatory diseases	[65]
p38 MAPK inhibitors	Preclinical	SB203580, Doramapimod, PH-797804	Potential off-target inhibition of hepatocyte stress resistance and regenerative pathways; narrow therapeutic window; systemic dose-limiting toxicity in clinical development for other diseases	[50]
Bruton’s tyrosine kinase (Btk) inhibitors	Preclinical (Theoretical for HIRI; Marketed for hematological malignancies)	Ibrutinib, Acalabrutinib, Zanubrutinib	Increased bleeding risk in clinical use; mild immunosuppression; low risk of severe hepatotoxicity at conventional doses	[138]
PAD4 inhibitors (Cl-amidine, GSK484, YW4-03)	Preclinical	Cl-amidine, GSK484, YW4-03	Favorable safety profile in preclinical studies; minimal impact on neutrophil phagocytic and antimicrobial functions; no severe systemic adverse events reported	[139,140,141]
PAD4 inhibitors (nanoparticle-encapsulated, e.g., Cl-amidine liposomes)	Preclinical	Cl-amidine liposomes, PAD4 inhibitor-loaded polymeric nanoparticles	Excellent safety profile in preclinical studies; reduced systemic exposure and off-target effects compared to free drug; no systemic immunosuppression reported	[142,143]
Recombinant human DNase I (rhDNase, Pulmozyme^®^)	Marketed (for cystic fibrosis; Phase II for acute pancreatitis/ARDS; Preclinical for HIRI)	Recombinant human DNase I (dornase alfa, Pulmozyme^®^)	Excellent safety profile in clinical use; no increased infection risk reported in short-term perioperative administration; no hepatotoxicity	[38,96,144,145,146]
Anti-histone H3/H4 neutralizing monoclonal antibodies	Preclinical	Anti-histone H3 monoclonal antibody, Anti-histone H4 neutralizing antibody	Theoretical risk of impaired nucleosome clearance and innate immune sensing; no severe adverse events reported in preclinical HIRI studies	[147]
LP342 (novel HDAC inhibitor)	Preclinical	LP342	Favorable safety profile in preclinical HIRI studies; no significant off-target toxicity reported at therapeutic doses	[148]
Sivelestat sodium (neutrophil elastase inhibitor)	Marketed (in Japan for acute lung injury; Preclinical for HIRI)	Sivelestat sodium hydrate	Mild and transient adverse events; minimal impact on neutrophil antimicrobial function in short-term perioperative use; low but non-negligible infection risk with long-term systemic administration	[149,150,151,152]
“NE-based nanovesicles”	Preclinical (Theoretical for HIRI; Preclinical in other inflammatory models)	Neutrophil elastase-targeted nanovesicles, NE-based biomimetic nanovesicles	Excellent biocompatibility in preclinical studies; minimal off-target distribution and systemic toxicity; no immunogenicity reported	[153]
Anti-MPO antibodies	Preclinical (Theoretical for HIRI; No clinical development for liver disease)	Anti-MPO neutralizing monoclonal antibody	Theoretical risk of impaired oxidative bactericidal function of neutrophils; no clinical safety data available	–
Anti-HMGB1 neutralizing antibodies	Preclinical	Anti-HMGB1 neutralizing monoclonal antibody	Mild immunosuppression; low risk of severe infection in short-term administration; no hepatotoxicity reported in preclinical studies	[70,154]

## 8. Conclusion and Future Directions

Despite substantial advances in hepatic ischemia–reperfusion injury (HIRI) research, critical knowledge gaps hinder the clinical translation of neutrophil-targeted therapies. Major challenges include the poorly defined heterogeneity and functional duality of neutrophils, the limited efficacy of single-target interventions, and the large translational gap between healthy young mouse models and clinical patients with comorbidities, aging, and sex-dependent differences.

Future research on neutrophil-targeted strategies for HIRI should focus on the following concrete and prioritized directions to overcome current translational bottlenecks and accelerate clinical application.

First, establishment of neutrophil-specific conditional gene knockout models. Conventional global knockout mice cannot distinguish cell-autonomous functions of core targets such as Syk, PI3Kγ, and PAD4 in neutrophils versus other liver resident cells. Construction of neutrophil-specific gene deletion models will help precisely clarify the independent role of these key molecules in driving HIRI, eliminate the interference of off-target cellular effects, and provide more reliable preclinical evidence for targeted therapy.

Second, development of PAD4 inhibitor-loaded neutrophil-targeted nanodelivery systems. Systemic administration of PAD4 inhibitors carries potential risks of impairing physiological antimicrobial defense. Nanoparticle-encapsulated and inflammation-targeted delivery systems can achieve precise enrichment at injured hepatic sites, reduce systemic drug exposure and immunosuppression risk, and improve the safety and efficacy of NETosis inhibition therapy.

Third, exploration and clinical validation of dynamic NET-related biomarkers. It is urgent to identify circulatory NET-derived markers that can dynamically reflect the timing and intensity of neutrophil activation and NET formation during HIRI. Such biomarkers will help define the optimal therapeutic time window, guide individualized intervention strategies, and enable rational design of subsequent clinical trials.

Fourth, preclinical translational advancement of isoform-selective PI3Kγ/δ inhibitors. Given the cell-specific functional paradox of the PI3K/Akt pathway, further preclinical studies should prioritize the efficacy and safety verification of immune cell-specific PI3Kγ/δ inhibitors. This strategy avoids the hepatotoxicity of non-selective pan-PI3K inhibition and represents a promising immuno-selective therapeutic direction for HIRI.

Ultimately, advancing HIRI treatment requires a shift toward systematic regulation of the entire inflammatory–thrombotic cascade, using clinically relevant humanized models and targeted delivery technologies. These strategies will enable precise control of neutrophil function, overcome translational barriers, and provide safe and effective perioperative hepatoprotection for liver transplantation and major hepatectomy.

## Data Availability

No new data were created or analyzed in this study. Data sharing is not applicable to this article.
